# Peptoid-Directed Formation of Five-Fold Twinned Au Nanostars through Particle Attachment and Facet Stabilization

**DOI:** 10.1002/anie.202201980

**Published:** 2022-03-15

**Authors:** Biao Jin, Feng Yan, Xin Qi, Bin Cai, Jinhui Tao, Xiaofeng Fu, Susheng Tan, Peijun Zhang, Jim Pfaendtner, Nada Y. Naser, François Baneyx, Xin Zhang, James J. DeYoreo, Chunlong Chen

**Affiliations:** Physical Sciences Division, Pacific Northwest National Laboratory 902 Battellt Boulevard, Richland, WA 99352 (USA); Physical Sciences Division, Pacific Northwest National Laboratory 902 Battellt Boulevard, Richland, WA 99352 (USA); School of Chemistry & Chemical Engineering, Linyi University The Middle Part of Shuangling Road, Linyi, Shandong Province, 276005 (China); Department of Chemical Engineering, University of Washington 1410 NE Campus Parkway, Seattle, WA 98195 (USA); Physical Sciences Division, Pacific Northwest National Laboratory 902 Battellt Boulevard, Richland, WA 99352 (USA); Physical Sciences Division, Pacific Northwest National Laboratory 902 Battellt Boulevard, Richland, WA 99352 (USA); Department of Biological Science, Florida State University 600 W College Ave, Tallahassee, FL 32306 (USA); Department of Electrical and Computer Engineering & Petersen Institute of Nanoscience and Engineering (PINSE) University of Pittsburgh 4200 Fifth Ave, Pittsburgh, PA 15260 (USA); Division of Structural Biology, Wellcome Trust Centre for Human Genetics, University of Oxford University Offices, Wellington Square, Oxford, OX1 2JD (UK); Diamond Light Source Harwell Science and Innovation Campus, Didcot OX11 0DE (UK); Physical Sciences Division, Pacific Northwest National Laboratory 902 Battellt Boulevard, Richland, WA 99352 (USA); Department of Chemical Engineering, University of Washington 1410 NE Campus Parkway, Seattle, WA 98195 (USA); Department of Chemical Engineering, University of Washington 1410 NE Campus Parkway, Seattle, WA 98195 (USA); Department of Chemical Engineering, University of Washington 1410 NE Campus Parkway, Seattle, WA 98195 (USA); Physical Sciences Division, Pacific Northwest National Laboratory 902 Battellt Boulevard, Richland, WA 99352 (USA); Physical Sciences Division, Pacific Northwest National Laboratory 902 Battellt Boulevard, Richland, WA 99352 (USA); Department of Materials Science and Engineering University of Washington 1410 NE Campus Parkway, Seattle, WA 98195 (USA); Physical Sciences Division, Pacific Northwest National Laboratory 902 Battellt Boulevard, Richland, WA 99352 (USA); Department of Chemical Engineering, University of Washington 1410 NE Campus Parkway, Seattle, WA 98195 (USA)

**Keywords:** Facet Stabilization, Five-Fold Twinned Au Star, Particle Attachment, Sequence-Defined Peptoids

## Abstract

While bio-inspired synthesis offers great potential for controlling nucleation and growth of inorganic particles, precisely tuning biomolecule–particle interactions is a long-standing challenge. Herein, we used variations in peptoid sequence to manipulate peptoid–Au interaction, leading to synthesis of concave five-fold twinned, five-pointed Au nanostars via a process of repeated particle attachment and facet stabilization. Ex situ and liquid-phase TEM observations show that a balance between particle attachment biased to occur near the star points, preferential growth along the [100] direction, and stabilization of (111) facets is critical to forming star-shaped particles. Molecular simulations predict that interaction strengths between peptoids and distinct Au facets differ significantly and thus can alter attachment kinetics and surface energies to form the stars. This work provides new insights into how sequence-defined ligands affect particle growth to regulate crystal morphology.

## Graphical Abstract

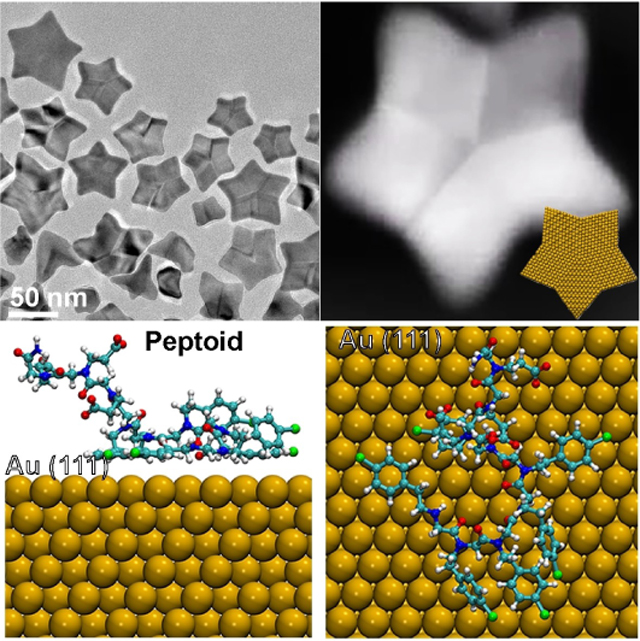

## Introduction

Achieving predictable synthesis of nanomaterials represents a long-standing goal in materials science. The use of organic ligands during collliodal synthesis of nanoparticles represents a widespread approach to achiveving this goal,^[1]^ because ligands can control both the size and morphology of the individual particles,^[2]^ as well as their collective interactions and assembly into 1D chains or 2D and 3D superlattices.^[3]^ The underlying sources of ligand-based control include altering interfacial free energies to define equilibrium shapes, inhibiting or enhancing facet-specific growth kinetics, and modifying interparticle potentials to direct assembly.^[4]^ For instance, attachment of Au nanoparticles can be driven to occur on different surfaces by introducing either CTAB or citrate as ligands.^[5]^ However, the range of control over growth kinetics, morphology and further assembly by simple organic ligands is limited, because the extent to which their structures and properties can be varied is constrained by their low chemical complexity. In contrast, proteins, peptides and other sequence-defined molecules offer the opportunity to introduce chemical moeities known to be effective in small-molecule ligands, but to do so within a far more diverse and easily engineered molecular context.^[4a,6]^ Recent efforts to synthesize inorganic nanomaterials using these sequence-defined ligands have shown great potential for achieving control over nucleation and growth of hierarchcial nanomaterials.^[6c,7]^ These studies have shown that the amphiphilicity of these ligands and their impact on particle attachment are significant for controlling the nanocrystal morphology.^[6a,b]^ For example, in a recent study by Ruan et al.,^[6a]^ peptides were reported to play a signficant role in the binding to specific facets of Pt nanocrystals and driving formation of multi-pods through oriented attachment of Pt clusters onto twin planes at the corners of Pt nanocubes.^[6a]^ Therefore, a better understanding of the sequence-specific control over ligand-ligand and ligand-particle interactions and their influences in particle attachment will significantly advance our ability to manipulate biomimetic nanocrystal synthesis.

Peptoids, which are a type of sequence-defined peptide mimetic, are particulary attractive for this purpose, because they are easy to synthesize and exhibit low structural complexity, extensive side-chain diversity and high stability.^[8]^ Unlike petpides and proteins, peptoids lack backbone hydrogen bond donors; thus they offer unique opportunities for tuning inter-molecular and molecule-particle interactions solely through the variation of side chain chemistry.^[7]^ Moreover, they have already proven to be a promising class of ligands for controlling inorganic crystal formation.^[6c]^

A wide-range of peptoids designed to mimic peptides through a “side chain similarity” approach have been developed to control inorganic crystallization.^[9]^ This approach has lead to control over crystal growth kinetics,^[9c,d]^ nanoparticle aggregation,^[10]^ and crystal habit either through facet stablization^[11]^ or enahncement of growth kinetics.^[10a]^ In one recent study, the choice of peptoid sequences in which the relative strengths of peptoid–Au and Au particle-Au particle interactions could be systematically tuned led to a balance between particle coasening and aggregation that resulted in novel highly-branched Au nanostructueres with unique plasmonic properties.^[6b]^ In that work, the hydrophilic monomer Nce [Nce=N-(2-carboxyethl)glycine] was chosen to mimic aspartic or glutamic amino acids and Ncp [Ncp= N-[2-(4-chlorophenyl)ethyl]glycines] was chosen to mimic hydrophobic and aromatic phenylalanine. The study demonstrated that tuning peptoid–peptoid and peptoid–Au NP interactions and peptoid amphiphilicity are crucial for driving particle attachment during the early stages of formation of the branched nanostructures.^[6b]^

**Figure 1.**
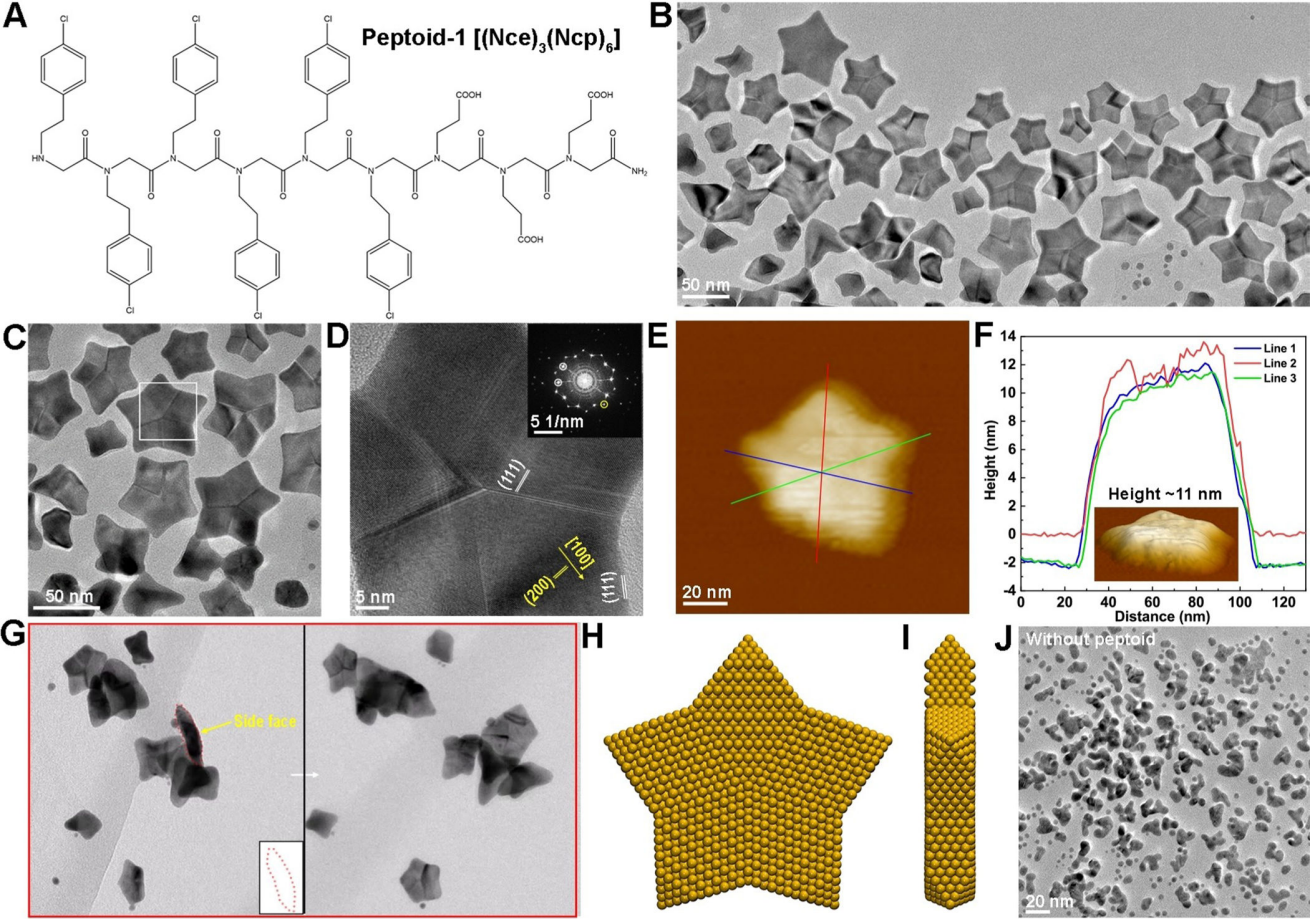
Structural characterizations of synthesized Au nanostars. A) The molecular structures of Pep-1, Nce=N-(2-carboxyethl)glycine, Ncp=N[2-(4-chlorophenyl)ethyl]glycine. B), C) Representative TEM image showing the five-twinned Au nanostar formed in the presence of 0.33 mM Pep-1, 1.64 mM HAuCl_4_ and 81.97 mM HEPES at PH 9.1. D) A HR-TEM image showing the five-twinned structure. E) AFM image of one Au nanostar. F) The line profiles showing the height of nanostar ≈11 nm. Inset is one surface plot of Au star. G) Electron tomography images of Au nanostars showing the near flat top and bottom surface. H), I) The proposed ideal star structure: top view (G) and side view (H), which shows the (011) basal facets and (111) side facets. J) The synthesized branched Au nanocrystals in the absence of peptoids.

Motivated by the importance of particle attachment in the biomimetic nanocrystal synthesis and inspired by our recent success in manipulating peptoid–peptoid and peptoid–particles surface interactions for controlling crystal growth kinetics^[12]^ and particle attachment,^[6b]^ herein, we reported peptoid-induced formation of novel five-fold twinned Au nanostars exhibiting five uniformly shaped star points. Four different peptoids were designed with systematic variations in the number Nce groups while maintaining a single hydrophobic domain Ncp (Figure 1A) and investigated their impact on Au particle formation dynamics and morphology. Our results show that a balance of the hydrophilic (Nce)_*n*_ (*n*=2, 3, 4, 6) and hydrophobic (Ncp)_6_ domains is important for peptoids to generate star-shaped Au particles (Figure 1B), the most mature of which exhibit five arms and five-fold twinning. Ex situ TEM and in situ liquid phase (LP) TEM investigations demonstrate that the Au stars form through particle attachement events combined with subsequent growth of the arms tuned through peptoid–Au interactions that stabilize (111) facets, thus promoting growth along the [100] directions. The complementary molecular dynamics (MD) simulations reveal the importance of facet-dependent peptoid–Au binding affinity in achieving this unique nanoparticle shape. By tuning peptoid–peptoid and peptoid–Au interactions, our results further show that peptoids play a significant role in controlling Au nanocrystal morphology by regulating both particle attachment and growth kinetics.

## Results and Discussion

Au nanoparticles were grown from peptoid-containing HAuCl_4_ solutions. HEPES buffer was used as a mild reducing reagent to initiate Au particle formation. Addition of a peptoid with three Nce groups (Figure 1A)—designated Pep-1—into the HEPES-HAuCl_4_ system led to formation of five-fold twinned nanostars with an average size of ≈62 nm (Figure 1B and Figure S2A), along with intermediate structures consisting of tetrapods and tripods (Figure S1A,B). A broad band at high wavelength (500–800 nm) in the UV/Vis spectra (Figure S2B) implied the formation of star-like particles based on previous literature.^[4c]^ TEM imaging shows that these star-shaped nanocrystals contain multiple twins (Figure 1C, D) that run from the center of the stars to the concave vertices of the points (Figure 1C, D and Figure S1C). The growth direction of the points lies along the [100] direction, while the side faces of each arm are assigned to be (111) facets when the stars are fully formed (Figure 1D and Figure S3). AFM imaging (Figure 1E, F and Figure S1C, D) and 3D TEM tomography (Figure 1G, Figure S4, Movie S1–S3) show that these five-fold twinned stars have nearly flat surfaces and a height of about 11–15 nm. Given that the (100) and (111) facets are perpendicular to the faces, we conclude that the faces must be close to (011) facets. Based on these directions, we derive the structure for mature Au nanostars shown in Figure 1H,I.

In peptoid-free control experiments, while some nanoparticle branching is observed, no star-shaped particles are formed regardless of solution pH values (Figure 1J and Figure S5), validating the critical role of Pep-1 in defining the unique morphology of these particles. More generally, comparison of these five-fold twinned particles to conventional five-fold twinned noble metal particles reported for synthetic procedures using other ligands^[13]^ reveals two morphological and structural distinctions. First, these particles are star shaped with flat faces instead of spheroidal-to-pentagonal. Second, the five twin boundaries are located at the concave vertices rather than at convex corners.

The morphological and structural distinctions of these star-shaped five-fold twinned particles from conventional five-fold twinned nanoparticles suggests their formation mechanism differs. Previous studies showed that the conventional five-fold twinned nanoparticles can either form through growth on pre-existing five-fold twinned seeds or by oriented attachment of untwineed primary particles followed by twinning to relax strain created by attachment.^[14]^ Notably, the twinning process in conventional five-fold twinned particles has been directly observed by in situ TEM during particle attachment.^[14c]^

First, we determined whether Au star formation occurs through a templating process due to pre-assembly of the peptoids into ordered structures with a 5-fold or star-shaped motif. We negatively-stained the reaction solution in the early stages of Au nucleation and found that Pep-1 itself failed to assemble into any ordered structure (Figure S6). Thus, we conclude that Pep-1 must act through its interactions with the Au stars during their formation, either by directing particle assembly or controlling facet-specific growth on seed particles or some combination of both. To determine which mechanism is at work, we investigated the early stages of Pep-1-induced Au nanostar formation using both ex situ TEM and in situ LP-TEM.

**Figure 2.**
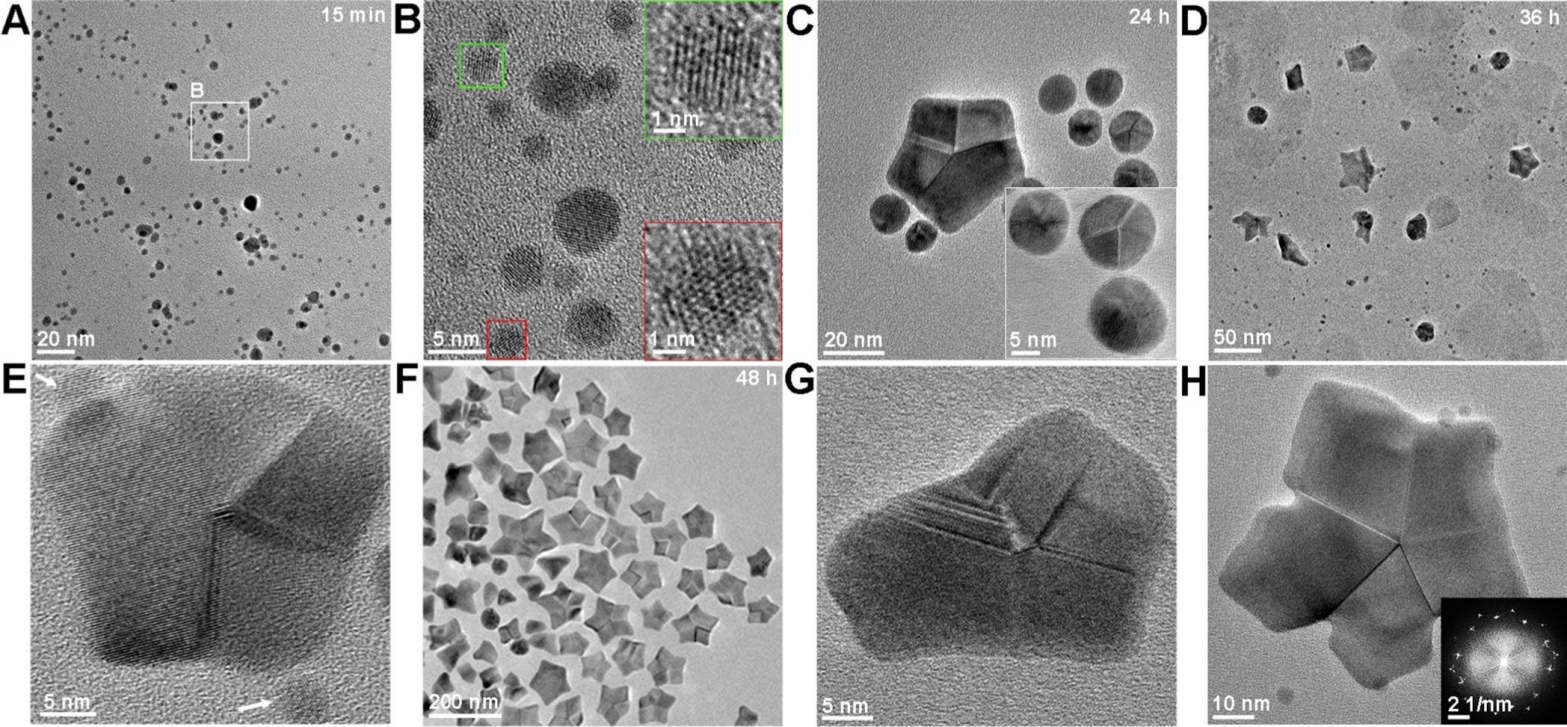
Time-dependent ex situ TEM images revealed some intermediate nanostructures during the Pep-1 induced formation of Au nanostars. A) TEM image showing Au nanocrystals formed at 15 min. B) HRTEM demonstrating the single-crystaline characteristics of small particles. C) TEM image of Au particles formed at 24 h, showing the formation of some spherical five-fold twinned nanocrystals. D) TEM image of Au nanocrystals formed at 36 h, showing the emergence of Au star. E) HRTEM image showing the five-fold twinned nanocrystals surrouded by small single crystalline particles. F) TEM image of Au nanocrystals formed at 48 h, showing the five-fold twinned star dominant structure. G), H) HRTEM image showing the exsitence of non-conventional or irregular five-fold twinned particles.

Ex situ TEM images were collected at a series of time points to monitor the intermediate structures that preceded the development of mature Au nanostars (Figure 2). The reduction of HAuCl_4_ precursor resulted in formation of ≈2–5 nm nanoparticles in 15 min (Figure 2A and Figure S7A), which were identified to be single crystal Au by HR-TEM (Figure 2B). By 24 h, we observed larger ≈10–15 nm spheroidal particles exhibiting five-fold twins, as well as the occasional larger pentagonal particle (Figure 2C). After 36 h of reaction, five-fold twined Au stars began to appear, but they were not yet fully formed and were within a mixture of the intermediate structures seen at the early time points (Figure 2D, E and Figure S7B). By 48 h, five-fold twinned Au stars were common, and many were fully mature, while nearly all of the small spheroidal particles had disappeared (Figure 2F) and partially formed stars still persisted (Figure 2G, H), some of which exhibited extremely concave vertices (Figure 2H) as previously observed in a star-shaped decahedron.^[15]^

**Figure 3.**
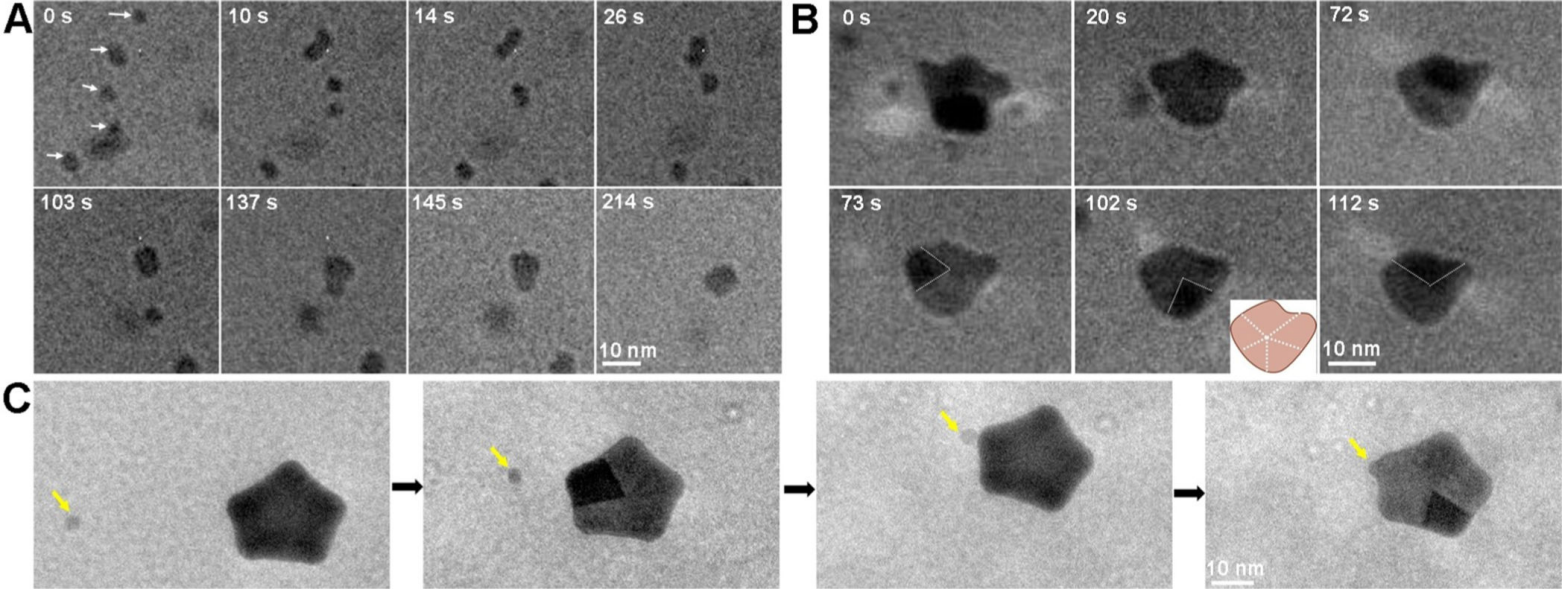
LP-TEM revealing the early stages of Au nanostar formation through particle attachment. A) The time dependent TEM images showing the attachment process of five individual nanoparticles into one big nanoparticle. B) The formation and growth of one five-twinned nanocrystal through particle attachment. Inset is the schematic of five-twinned structure. C) The further growth of regular five-twinned nanocrystal into concave Au star by an attachment of small nanocrystal (marked by yellow arrow) on the corners. Red arrow highlights the sharp corner. Electron dose rates are 51.1 eÅ^−2^s^−1^ (A), 51.1 eÅ^−2^s^−1^ (B), and 41.4 eÅ^−2^s^−1^ (C) respectively. The definition of zero time point is when the TEM images are captured, which is ≈20 min after preparation of reaction solutions.

To understand how the five-fold twinned stars evolve, we next used in situ LP-TEM, which has been emplyed extensively to investigate nanoparticle formation mechanisms due to its high temporal and spatial resolution.^[16]^ By sealing initially pre-reacted solutions containing the already formed spheroidal single crystalline Au nanoparticles, HEPES and Pep-1 into liquid-cell, we directly observed particle growth and attachment processes (Movie S4). Upon imaging, we found that many ≈5 nm nanoparticles were already present in the liquid-cell (Figure 3A–0s) and we observed no new nucleation events, showing that HEPEs had already reduced the concentration of solute ions below the level needed to nucleate new particles prior to imaging. As time progressed, particle attachment events dominated the growth prcoess, rather than Oswald ripeening caused by the solubility difference between particles of different sizes (Figure 3A). Imaging of five Au nanoparticles moving freely in the solution reveals repeated attachment events until they have all coalesced into a single larger (≈10 nm) particle (Figure 3A). Based on the particle diameters, the final particle volume is found to be approximately equal to the sum of the initial particle volumes. Though we cannot determine from the in situ images whether or not the coalesced particles are twinned, we assunme they are based on their size and comparison to the ex situ TEM results shown above, as well a recent in situ TEM study of five-fold twinned Au nanoparticle formation in vacuum.^[14c]^ In that study, repeated attachment events followed by decomposition of high-energy grain boundaries to form twins resulted in the five-fold twinned structure of the final particles. The much lower values of interfacial energies and heats of solvation in solution as compared to surface energies and heats of vaporization in vacuum, may provide the higher mobility needed to enable the process to occur at room temperature in aqueous solution.

As time progressed, we observed further particle aggregtion events resulting in formation of multi-twinned nanocrystals with the beginnings of the star points (Figure 3B, Movie S5). The rotation of the nanocrystal in the solution enabled us to distinguish several different grains and determine that it was, indeed, a five-fold twinned nanocrystal. Notably, following the attachment events, the nanocrystal morphology partially relaxed towards a more compact shape either by atomic rearrangement within the crystal or via surface diffusion, suggesting that, at this size, the points observed at later times are not yet stable. However, this relaxation process may be acelerated by the energy from the electron beam and whether it occurs in the absence of the beam is unknown.

As the five-fold twinned particles continued to grow in size through these repeated aggregation events, they developed the five points and concave vertices characteristic of the final star-shaped particles (Figure 3C, Movie S6). The extension of the points was aided by attachment events and, in fact, attachement tended to occur preferentially on the outward-facing protrusions (Figure 3C). The combination of ex situ and in situ TEM observations lead us to deduce the following pathway for formation of the five-fold twinned Au nanostars: 1) the attachment of multiple ≈2–5 nm single crystal particles results in formation of five-fold twinned spheroidal particles ≈10–15 nm in size. 2) The particles continue to grow through repeated attachment events, relaxing towards an equilibrium shape after each event. 3) As the size exceeds ≈20 nm, attachment and relaxation begins to favor extension along the [100] directions. 4) Once the sides of the arms reach the (111) facet direction at a particle size of ≈40–50 nm, the shape is stabilized and further addition only increases the size of the stars, but does not alter the shape.

Given that the five-fold twinned Au nanoparticles are not unique to the peptoid–HAuCl_4_ system, but the star points are, at a minimum we can conclude that Pep-1 modulates the surface energies to promote growth along the [100] direction and stabilize the (111) facets. In addition, the results show that Pep-1 promotes the relaxation process, because, in its absence, attachment events result in randomly branched particles that do not relax towards a compact shape (Figure 1J). Finally, as the particles grow in size, Pep-1 appears to bias attachment to occur at the (100)-facing protrusions. A similar effect was reported for peptide-based ligands that were selected to bind to specific facets of Pt and resulted in growth of tetrapods by attachment of primary particles onto the corner facets.^[6a]^ However, in peptoid-free solutions, five-fold twinning of metal nanoparticles only reduces the energy when particles are small. Once they exceed about 14 nm,^[14c]^ the equilibrium structure is that of compact, twin-free single crystals. Consequently, the star shape obtained with Pep-1 must eventually represent a metastable shape with the global minimum defined by a single crystal bounded by (111) facets. However, once the particles reach the size at which the points develop, there is little driving force for this shape transition, either through internal atomic rearrangements or dissolution and redeposition, because the local facet directions are already low energy. Hence the particles remain star-shaped (Figure S8).

To understand the effect of specific experimental conditions on the ability of Pep-1 to guide the formation process, we performed a series of experiments in which we varied solution parameters, including Pep-1 and HAuCl_4_ concentrations, and the HEPES buffer pH value. These parameters should alter the Pep-1 coverage on the Au nanoparticles either by changing their relative ratios or the degree of deprotonation of Pep-1, respectively. The latter effects the degree to which the carboxyl groups of peptoids interact with Au surfaces through electrostatic interactions.^[17]^

**Figure 4.**
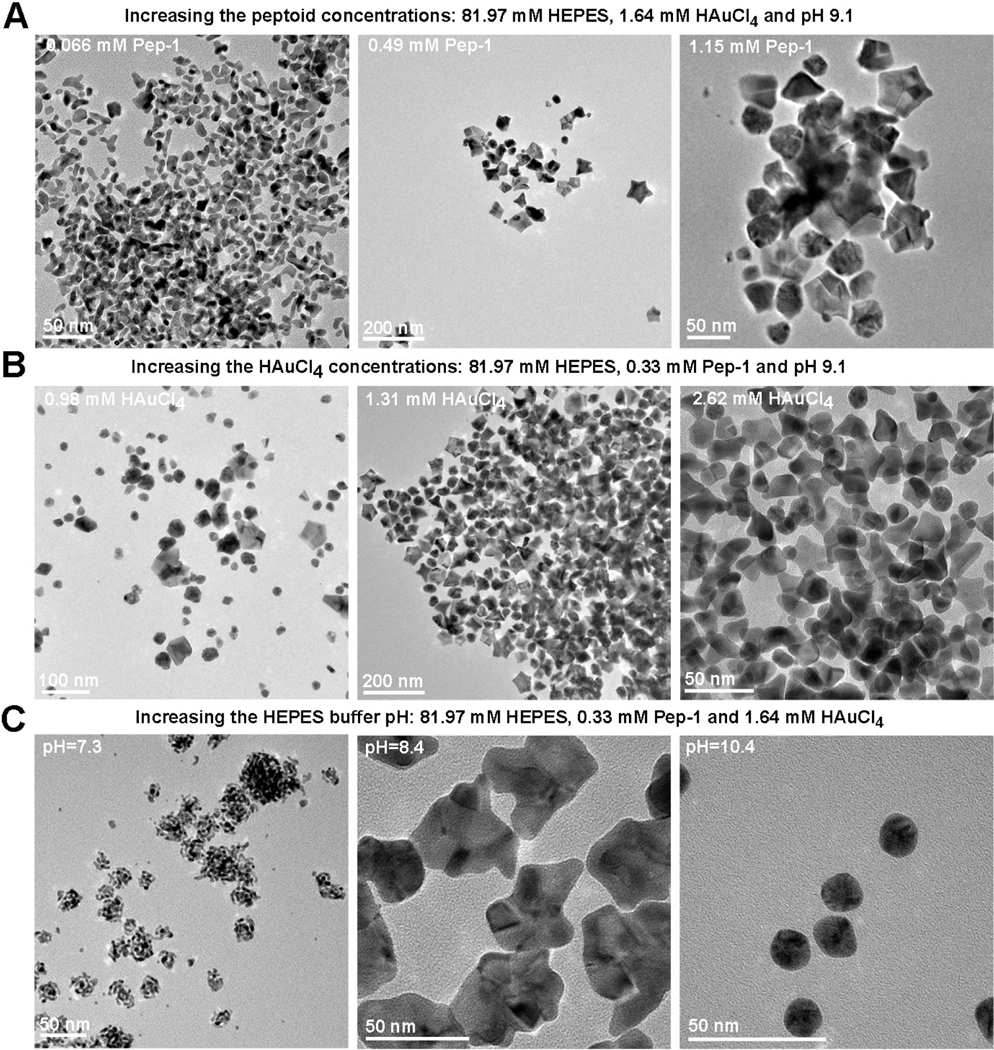
The TEM images showing the formation of various Au nanostructures in the specific condition, where all conditions are same to those in Figure 1C except for one varied experimental parameter: peptoid concentrations (A), HAuCl_4_ concentrations (B) and the HEPES buffer solution pH (C).

The results show that, when the Pep-1 concentration is increased, the resulting nanocrystal shapes initially evolve from randomly branched structures to five-fold twinned Au stars (Figure 4A), but at sufficiently high Pep-1 concentrations, star shaped particles are less common. The initial concentration of HAuCl_4_ also exhibits a significant effect on morphology (Figure 4B), with the occurrence of well-formed stars common at 1.31 mM and 1.64 mM (Figure 1B, Figure 4B) lost at both high (2.62 mM) and low (0.98 mM) concentration. Both the change of Pep-1 and HAuCl_4_ concentrations changed particle behaviors in solution through kinetics control. Less pep-1 adsorbed onto surface results in the formation of branched structure, presumably through random attachment during irregular Au nanocrystal process.^[6b]^ Solution pH, is equally powerful in regulating the effect of Pep-1 (Figure 4C). At pH 7.3, only small, highly branched particles are observed. At pH 8.4 were observed, the particles become larger and more compact, but the branching is still irregular. Only at pH 9.1, well-developed stars are observed (Figure 1B), while at a still higher pH value 10.4, the particles remain at the stage of spheroidal twinned structures.

These results suggest that a fine balance between attachment rates, surface energies and relaxation kinetics are needed to achieve the five-fold twinned structure and the (111) faceted arms that define the nanostars. This balance can be rationalized in terms of absolute and relative coverages of Pep-1 on the (111) and (100) facets. Adequate Pep-1-to nanoparticle ratios (Pep-1 vs HAuCl_4_ concentrations) and interaction strengths (deprotonation) are required to promote attachment events, relaxation towards equilibrium, and (111) stabilization. If either is too low, then relaxation from a randomly branched structure that forms in the absence of Pep-1 does not occur. However, with too strong an interaction strength or too high a Pep-1-to nanoparticle ratio, the coverage on the (100) faces may become too high, thereby inhibiting both attachment at the (100) protrusions and growth along the [100] directions to form the arms of the star.

**Figure 5.**
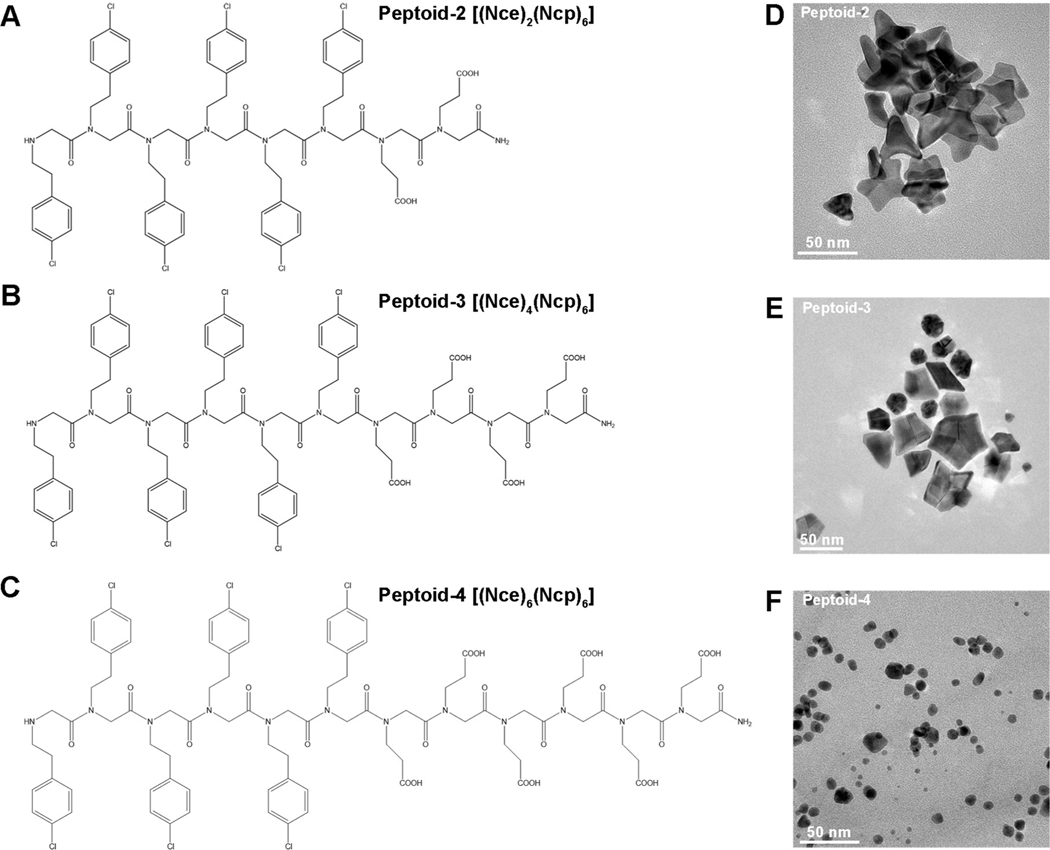
Nanostructures induced by peptoids with varied numbers of Nce groups. A)–C) The structures of peptoids: Pep-2–Pep-4. Nce=N-(2-carboxyethl)glycine, Ncp=N-[2-(4-chlorophenyl)ethyl]glycines. D)–F) TEM images of Au nanocrystals induced by Pep-2 (D), Pep-3 (E), Pep-4 (F) at pH 9.1.

The effect of deprotonation implies that the number of peptoid carboxyl groups, which control the strength of the peptoid–Au electrostatic interaction by tuning the amphiphilicity and surface charge of peptoids,^[6b]^ should be an important factor in determining the capacity of a peptoid to drive formation of star-shaped particles. To investigate the importance of this parameter, we synthesized an additional three peptoids (Pep-2 to Pep-4) in which we systematically varied the number of Nce groups from the original three found in Pep-1 while keeping the hydrophobic domain unchanged (Figure 5A–C). We found that Pep-2 and Pep-3, with two and four Nce groups, respectively, can also produce Au star-like particles but with lower yields than obtained with Pep-1 (Figure 5D,E). In the case of Pep-2, which has the fewest carboxyl groups, the branches tend to be elongated and irregular, again suggesting the occurrence of particle attachment events without adequate relaxation towards uniform branch angles. While in the case of Pep-3, which has one more carboxyl group than Pep-1, there appear to be many more immature particles hinting at a more infrequent occurrence of particle attachment events at the (100) protrusions and/or growth along the [100] directions to form the arms of the star. This trend continues with Pep-4, which has the largest number of carboxyl group and only induces the formation of near-spherical multi-twinned nanoparticles of greater number and smaller size (Figure 5F), implying a much lower frequency of particle attachment events and/or a lack of preferential growth along the [100]. These changes in morphology from the star-like shapes formed in the presence of Pep-1 (Figure 1C), demonstrate the sequence-dependence of the peptoid–Au interactions and, consequently, particle attachment rates, surface energies and relaxation kinetics, either because of changes in the inherent peptoid–Au interactions or the resulting absolute and relative peptoid coverages on the particle surfaces.

While it is difficult to assess how a change in the number of Nce groups affects particle attachment or relaxation directly from experiment, computational means can provide constructive insights at the molecular level. Here, we compare two cases where well-defined star shapes (Pep-1) and nearly spherical shapes are formed (Pep-4). In our separate computational work,^[18]^ we calculated the adsorption free energy of Pep-1 on Au(100) and Au(111) using atomistic MD simulations with parallel-bias metadynamics (PBMetaD)^[19]^ and identified the origin of facet selectivity of Pep-1 to Au. In that work, we showed that the binding affinity of Pep-1 on Au(111) is nearly 12-fold compared to that on Au(100). This difference can be attributed to a collective effect of both the side chain selectivity and the interfacial water network that protects the surface from molecule adsorption. Specifically, the aromatic group on the Ncp side chain strongly favors Au(111) over Au(100). And the adsorption affinity of the ring on Au(111) can be further enhanced by maximizing the direct contact achieved by laying flat on the surface. This configuration is obtainable with these peptoids as allowed by the flexibility of both the backbone dihedrals and the side chain connection to the backbone. In contrast, the deprotonated carboxyl groups of Nce side chains show very weak and non-selective binding to both facets, thus rationalizing the observation of spherical particles at high pH (Figure 4C). In addition, water molecules form the more ordered, stagnant layer on Au(100) further protecting the facet from general molecular adsorption.

**Figure 6.**
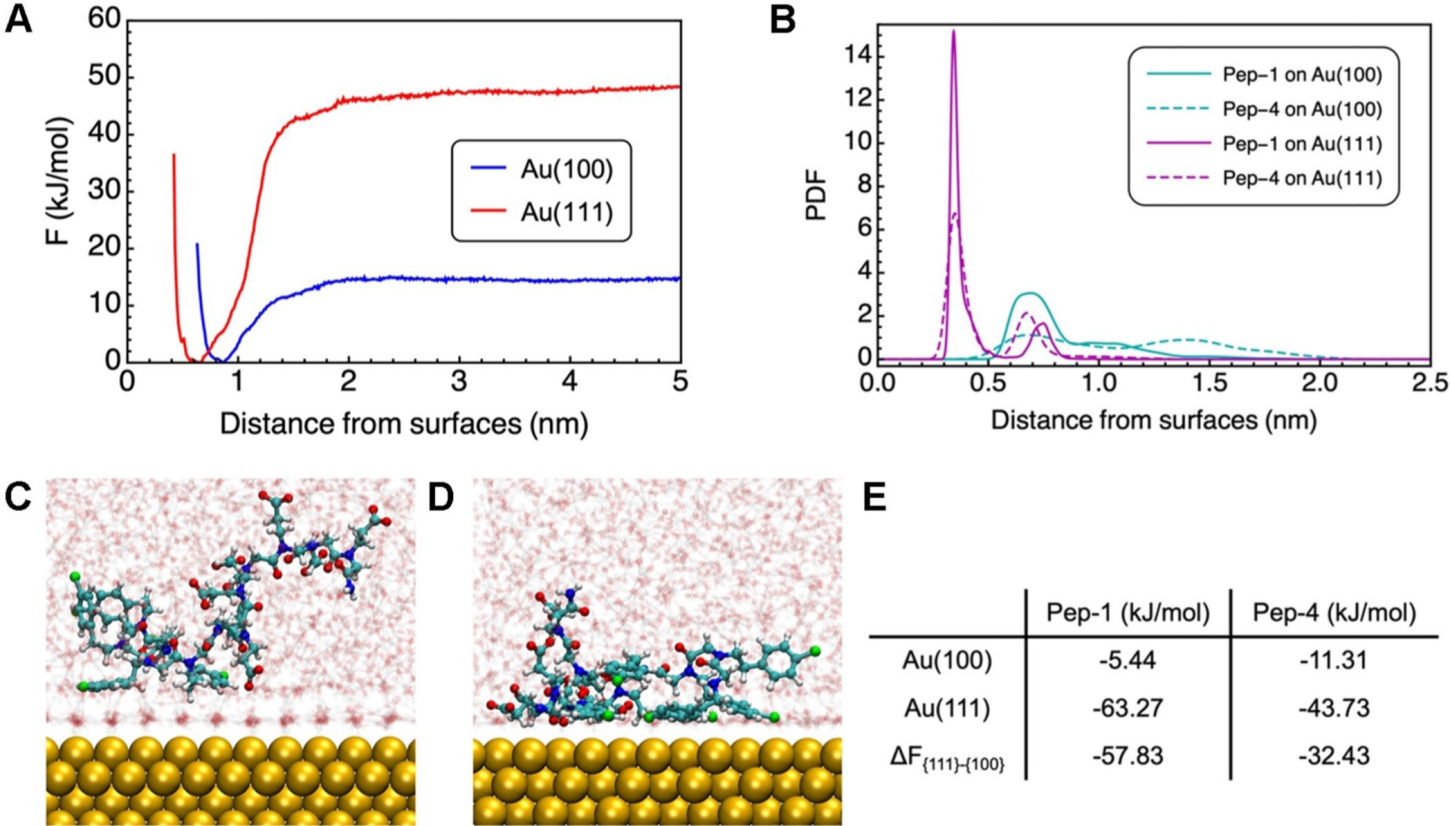
Computational studies of selected peptoids on Au surfaces. A) The adsorption free energy of the COM of Pep-4 on Au(100) and Au(111) calculated using PBMetaD. The x-axis indicates the orthogonal distance between the center of mass of Pep-4 and the surface that is represented by the average position of the top layer Au atoms. B) The probability distribution function of the center of mass of the aromatic rings orthogonal to Au surfaces. C), D) The side views of Pep-4 on Au(100) and Au(111) from unbiased MD simulations. Atoms are shown in the following color schemes: Au (golden), carbon (cyan), oxygen (red), nitrogen (blue), chlorine (green), and hydrogen (white). Water molecules are translucent for clarity. E) The adsorption free energy of peptoids on Au(100) and Au(111), as well as the relative difference between the two facets. Values for Pep-1 are adopted from previous work.

Here, we take a similar approach and examine how the adsorption behavior or selectivity is different for Pep-4 as compared to Pep-1. We constructed two simulation systems in which we solvated a single Pep-4 chain in water near Au(100) and Au(111), respectively. All carboxyl groups on Pep-4 were deprotonated to accommodate the basic solution pH, and Na^+^ and Cl^−^ ions were added to mimic the ionic environment. After extensive equilibration, we performed PBMetaD to calculate the adsorption free energy of Pep-4 at the water-Au interfaces (Figure 6A). Although Pep-4 still exhibits the same trend in facet-selectivity [i.e., favoring Au(111)], the relative preference is largely reduced from Pep-1 (i.e., from a 12-fold preference to a 4-fold preference) (Figure 6E). Correspondingly, our surface plasmon resonance (SPR) measurements (Figure S9) show that Pep-1 exhibits a stronger binding affinity for Au than Pep-4 does. The change in relative facet selectivity arises from the amphiphilicity and can be rationalized from adsorption configuration. We find an obvious preference of the nonadsorbing hydrophilic Nce side chains to the solution phase than the solid–liquid interface (Figure 6C, D) inevitably lifting up the Ncp segments and consequently weakening the favored interaction between the Ncp side chains and Au(111). The “lifting” effect from the Nce sidechains to the Ncp sidechains are quantified by comparing the density distribution function of the center of mass (COM) of the aromatic rings on Ncp for Pep-1 and Pep-4, (Figure 6B). For both surfaces, the density peaks of the COM for Pep-4 (dashed lines) are lowered and shifted away from the surface.

As discussed above, the morphological control in five-fold twinned nanostar formation through particle attachment requires not only surface stablization to counterbalance the energy gain associated with the twin plane formation, but also possible kinetic regulation in growing the star tips. Based on the significant difference in relative facet preference between Pep-1 and Pep-4, we argue that the thermodynamic influence from the peptoid in passivating Au(111) plays an important role in stablizing the five twin planes, which is largely weakened for Pep-4. At the same time, the much weaker binding to the (100) should result in low coverage to promote attachment at the protrusions and render it a higher energy face than (111), thus promoting atom attachment along that direction. How the peptoids then impact the ease of relaxation towards the equilibrium shape remains unclear as we cannot tell whether atomic transport occurs aong the surface or via the solution.

To further highlight the critical roles of peptoid-induced particle attachment kinetics and facet-specific stability in the formation of Au stars, we performed in situ UV/Vis^[20,17a]^ and inductively coupled plasma optical emission spectrometry (ICP-OES) experiments to monitor the changes of reactions in the presence of Pep-1 or Pep-4, or without addition of peptoids. UV/Vis measurements, which have been frequently used previously to evaluate the reduction kinetics of gold salts in the presence of additives,^[20]^ suggested that Pep-1 and Pep-4 exhibited similar reduction kinetics (Figure S10). ICP-OES results showed that these three systems exhibited almost indistinguishable changes in the remaining Au^III^ species in the reaction solution as a function of reaction time (Figure S11), suggesting that the reduction kinetics of gold salts is not a key factor leading to the Pep-1-induced formation Au nanostars. This conclusion is consistent with numerous previous studies of Au nanoparticle formation.^[20a,21]^ Rather, this phenomenon arises from changes in atom attachment kinetics and facet stabilization due to facet-specific binding affinity, which in turn depends on peptoid sequence.

## Conclusion

In summary, we have discovered a peptoid sequence that produces a unique Au nanoparticle morphology conmsisting of five-fold twinned Au stars with arms pointing along the [100] direction and bounded by (111) side facets when fully mature. Through careful ex situ TEM and in situ LP-TEM investigations, we deduced a stepwise formation mechanism: the intitial formation of small single crystals, followed by their attachment for producing five-fold twinned spherical particles, and then further attachment and relaxztion to form the well-developed five-fold twinned stars. This process depends on a strong energetic preference of peptoid binding to Au(111) over Au(100), as demonstrated by our simulations, and is strongly influenced by the ratio of peptoid-to-Au and the absolute and relative strengths of peptoid binding to Au faces as determined by the number of carboxyl side chains and the degree of protonation. These factors act to define a balance between the rate and location of particle attachment events, the kinetics of relaxation towards equilibrium, and (111) stabilization. In general, this work shows that control over peptoid–particle interactions provides diverse possibilities for developing new functional nanomaterials for a range of applications.

## Supplementary Material

supplementary materials

tomography tilt series

## Data Availability

The data that support the findings of this study are available in the Supporting Information of this article.
