# Supplementary material for: Peptoid-Directed Formation of Five-Fold Twinned Au Nanostars through Particle Attachment and Facet Stabilization
Source: Angew Chem Int Ed Engl. Author manuscript; Available in PMC 2022 Sep 28. (PMC9258440; doi:10.1002/anie.202201980)
Supplement: supplementary materials [file NIHMS1813863-supplement-supplementary_materials.pdf]

Peptoid-Directed Formation of Five-Fold Twinned Au Nanostars through Particle Attachment and Facet Stabilization

Biao Jin,‡ [a] Feng Yan, ‡ [a, b] Xin Qi, [c] Bin Cai,[a] Jinhui Tao,[a] Xiaofeng Fu,[d] Susheng Tan,[e] Peijun Zhang,[f, g] Jim Pfaendtner, [c, a] Nada Y. Naser, [c] Francois Baneyx, [c] James J DeYoreo,*[a, h] and Chun-Long Chen*[a, c]

[a] Dr. B. Jin, Dr. F. Yan, Dr. B. Cai, Dr. J.H. Tao, Dr. J. Pfaendtner, Dr. J.J. DeYoreo, Dr. C.L. Chen
Physical Sciences Division
Pacific Northwest National Laboratory
902 Battellt Boulevard, Richland, WA 99352, USA
E-mail: James.DeYoreo@pnnl.gov, Chunlong.Chen@pnnl.gov.

[b] Dr. F. Yan
School of Chemistry & Chemical Engineering
Linyi University
The Middle Part of Shuangling Road, Linyi, Shandong Province, 276005, China

[c] Dr. X. Qi, Dr. J. Pfaendtner, N. Y. Naser, Dr. F. Baneyx, Dr. C.L. Chen
Department of Chemical Engineering
University of Washington
1410 NE Campus Parkway, Seattle, WA 98195, USA

[d] Dr. X. Fu
Department of Biological Science
Florida State University
600 W College Ave, Tallahassee, FL 32306, USA

[e] Dr. S.S Tan
Department of Electrical and Computer Engineering & Petersen Institute of Nanoscience and Engineering (PINSE)
University of Pittsburgh
Pittsburgh, PA 15261, USA
4200 Fifth Ave, Pittsburgh, PA 15260, USA

[f] Dr. P. Zhang
Division of Structural Biology, Wellcome Trust Centre for Human Genetics
University of Oxford
University Offices, Wellington Square, Oxford, OX1 2JD, UK

[g] Dr. P. Zhang
Diamond Light Source
Harwell Science and Innovation Campus
Didcot OX11 0DE, UK

[h] Dr. J.J. DeYoreo
Department of Materials Science and Engineering
University of Washington
1410 NE Campus Parkway, Seattle, WA 98195, USA

[‡] These authors contributed equally to this work.

Supporting Figures S1−S10 as follows; peptoid synthesis methods and detailed structural characteristics of the Au star (PDF).

Movie S1: TEM tomography shows the 3D structure of Au star (AVI).

Movie S2: STEM tomography shows the 3D structure of Au star (AVI).

Movie S3: STEM tomography and reconstructed 3D structure for Au star (AVI).

Movie S4: LP-TEM reveals the particle attachment for nanocrystal growth (AVI).

Movie S5: LP-TEM reveals the particle attachment for five-twinned particle formation (AVI).

Movie S6: LP-TEM shows that one particle attached onto the corner of star (AVI).

Peptoid Synthesis

All peptoids were synthesized on a commercial Aapptec Apex 396 robotic synthesizer on using a solid-phase submonomer cycle as described previously. *(1, 2)* All amine submonomers and other reagents used for our peptoids synthesis are obtained from commercial sources and used without further purification. Rink amide resin (0.52 mmol/g, AappTec) was used to generate C-terminal amide peptoids. In this method, the Fmoc group on the resin was deprotected by adding 2 mL of 20% (v/v) 4-Methylpiperidine/N,N-dimethylformamide (DMF), agitated for 20 min, drained, and washed with DMF. All DMF washes consisted of the addition of 1.5 mL of DMF, followed by agitation for 1 min (repeated five times). An acylation reaction was then performed on the amino resin by the addition of 1.6 mL of 0.6 M bromoacetic acid in DMF, followed by 0.35 mL of 50% (v/v) N,N-diisopropylcarbodiimide (DIC)/DMF. The mixture was agitated for 30 min at room temperature, drained, and washed with DMF. Nucleophilic displacement of the bromide with various primary amines occurred by a 1.6 mL addition of the primary amine monomer as a 0.6 M solution in N-methyl-2-pyrrolidone (NMP), followed by agitation for 60 min at room temperature. The monomer solution was drained from the resin, and the resin was washed with DMF as described above. The acylation and displacement steps were repeated until a polypeptoid of the desired length was synthesized. All reactions were performed at room temperature. Peptoid chains were cleaved from the resin by addition of 2.0 mL 95% (v/v) trifluoroacetic acid (TFA) in water for 35 min, which was then evaporated off under a stream of nitrogen gas. Following cleavage, peptoids were dissolved in 4.0 mL mixture (v/v = 1:1) of water and acetonitrile for further purification.

All peptoids were purified by reverse-phase HPLC on a XBridge^{TM} Prep C18 10 μm OBD^{TM} (10 μm, 19 mm × 100 mm), using a gradient of 5-95% acetonitrile in H_2O with 0.5% TFA over 15 min. The final products were analyzed using Waters ACQUITY reverse-phase UPLC (5–95% CH_3CN in H_2O at 0.4 mL/min over 5 min at 40°C with a ACQUITY®BEH C18, 1.7 μm, 2.1 mm × 50 mm column) that was connected with a Waters SQD2 mass spectrometry system. The final peptoid products were lyophilized at least twice from their solution in mixture (v/v = 1:1) of water and acetonitrile. All lyophilized peptoids were finally divided into small portions (3.0 × 10^{-6} mol) and stored at -80°C.

Information of Peptoid Sequences

Structures of the synthesized peptoids and molecular weight of each peptoid as determined UPLC-MS are shown below.

Peptoid-1 Nce_3Ncp_6: 1578.25 (Molecular Weight), 1578.75(Found).

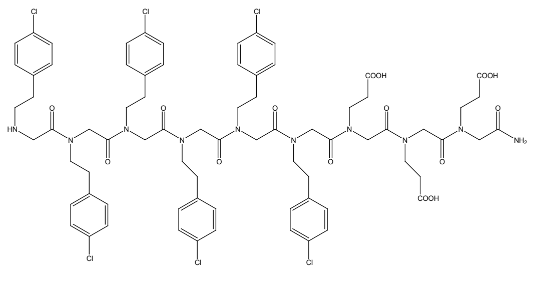

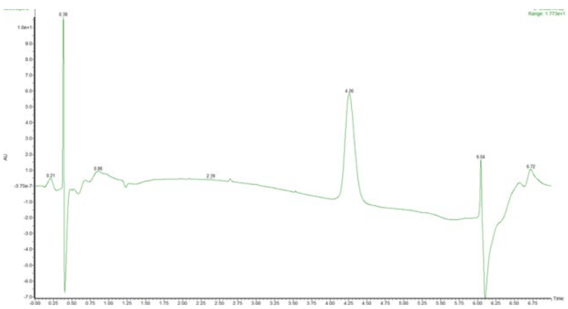

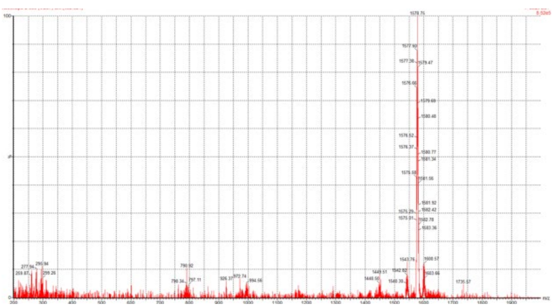

Peptoid-2 Nce_2Ncp_6: 1449.14 (Molecular Weight), 1448.57 (Found).

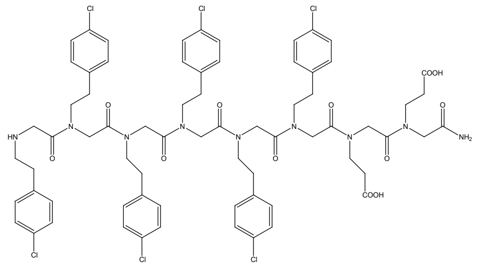

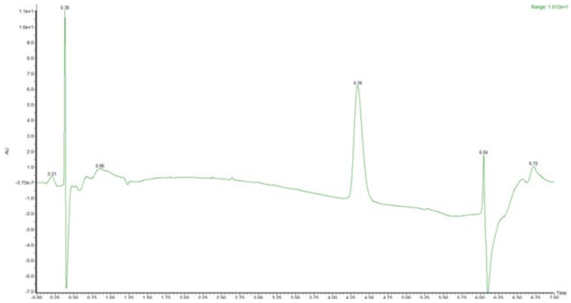

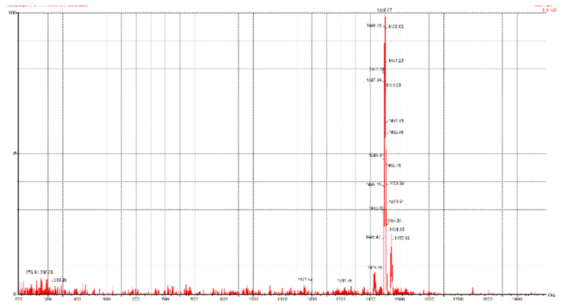

Peptoid-3 Nce_4Ncp_6: 1707.37 (Molecular Weight), 1708.13 (Found).

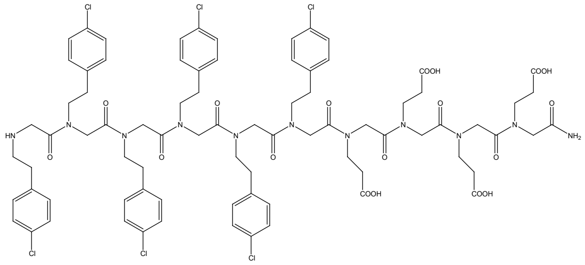

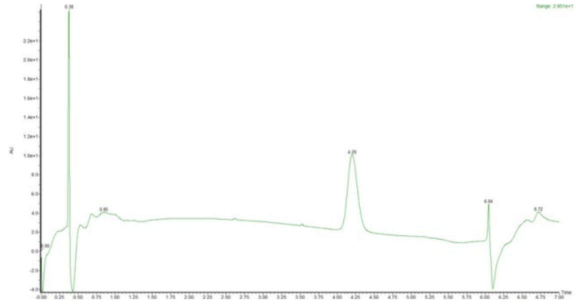

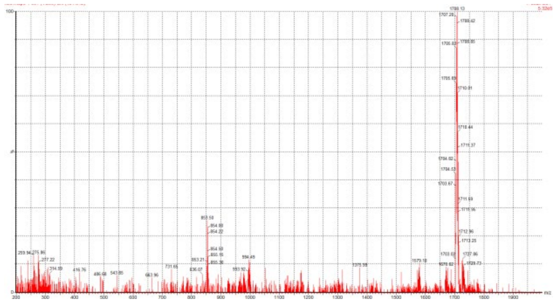

Peptoid-4 Nce_6Ncp_6: 1965.60 (Molecular Weight), 983.62 (Found: $[M/2+H^+]$).

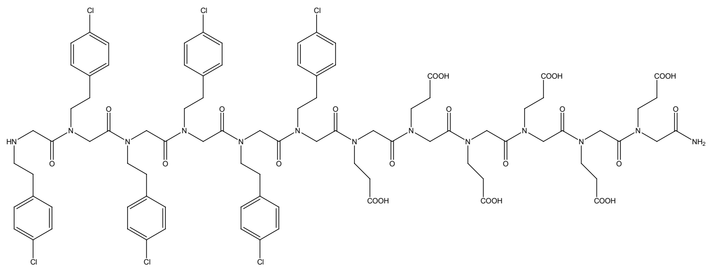

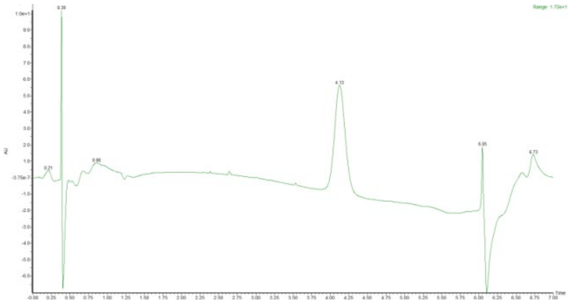

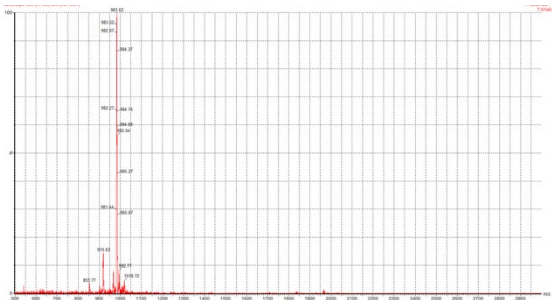

Supporting Figures

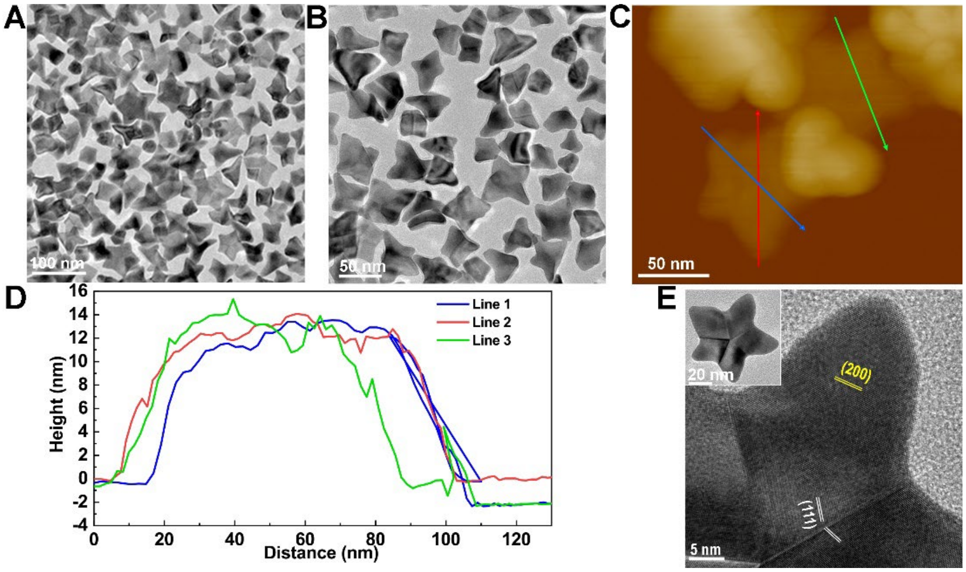

Figure S1. Characterizations of Au nanostars induced by Pep-1. (A-B) TEM images of Au stars. (C) The AFM image of two Au stars. (D) The line profiles along three different directions of two Au stars. (E) TEM image showing one five-fold twinned star with very sharp corners.

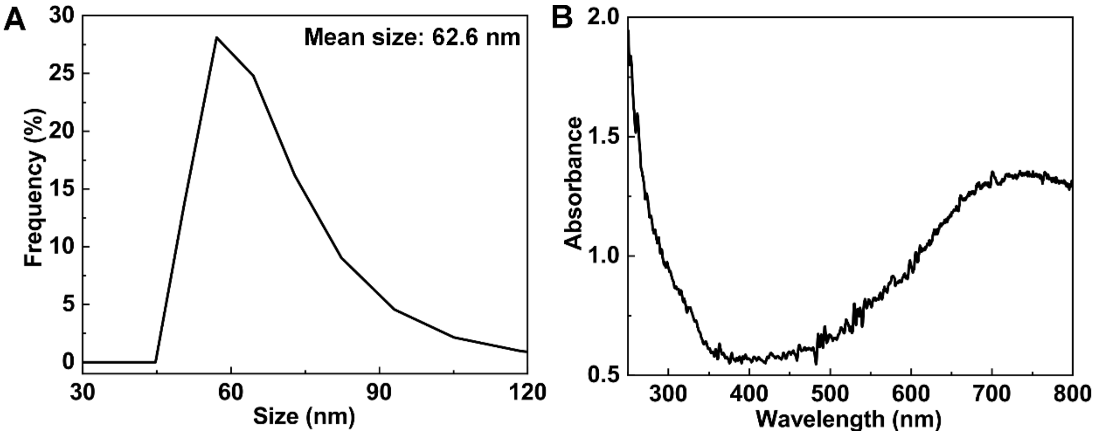

Figure S2. Size and optical properties of Au star solution at ensemble-averaged level. (A) The DLS showed a broad size distribution of Au particles in solution. (B) Ensemble UV−vis absorption spectra showed that a broad band at higher wavelengths (600 nm-800 nm) appeared and is attributed to the plasmon resonance along the long axes of the five arms, which is similar to previous work for star-like metal particle.[3] The origin of the broad plasmon resonance is not clear from the ensemble spectrum, as it can be a consequence of polydispersity in size/shape, aggregation of star, or intrinsic to each Au star.

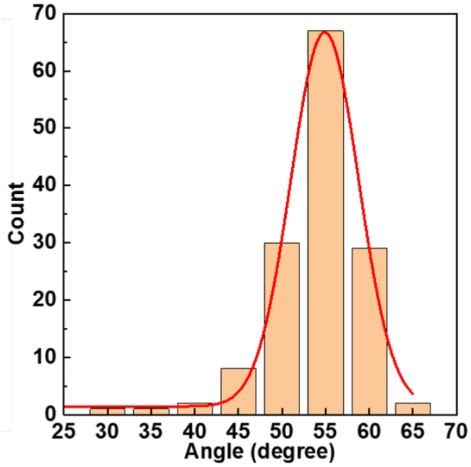

Figure S3. The estimated angle between the normal to edges of Au stars in Figure 1B and the [100] direction. The plot shows an average angle of ~55°, indicating that the side face of Au starts is along [111] direction.

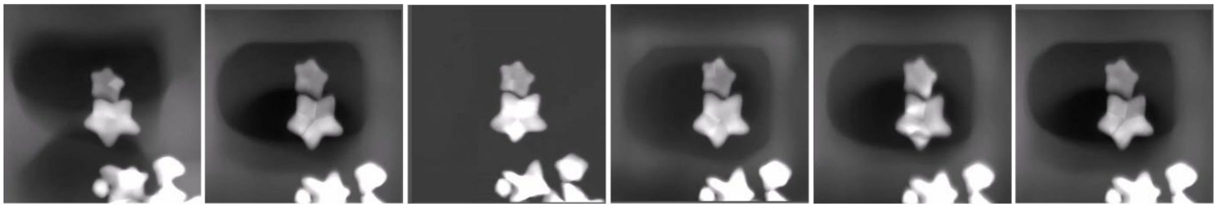

Figure S4. Electron tomography images of Au stars from a +/-30° tilt series with a step size of 1° . Examples of projection images taken in HAADF-STEM from left to right with tilt angle: -30°, -20°, 0°, 10°, 15° and 20°.

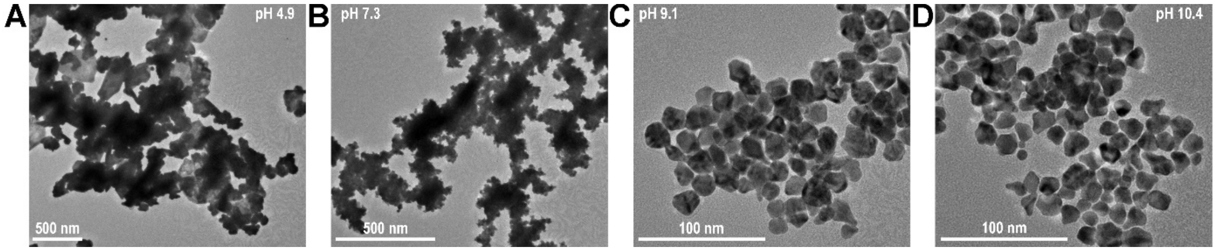

Figure S5. The Au particles with various morphologies were prepared at different pH values without peptoid introduction.

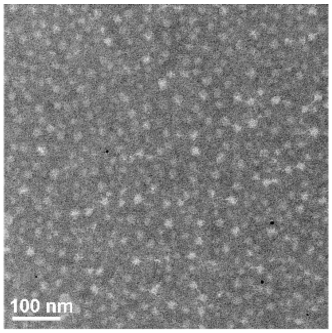

Figure S6. Negatively stained TEM image showing that no ordered Pep-1 assemblies were formed in the early stage of Pep-1-induced Au nanocrystals formation. 2% phosphotungstic acid was used for negative staining of the reaction solution in the early stage.

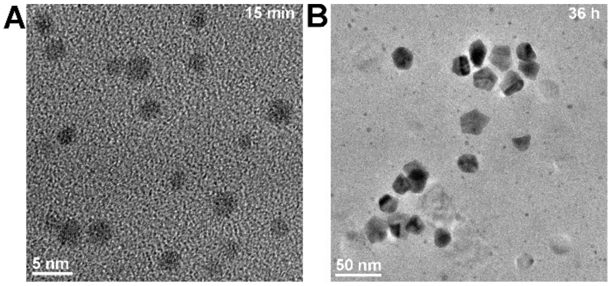

Figure S7. The Au nanostructures at different stages of Pep-1-induced Au star formation. (A) HRTEM shows the single crystaline characteristics of small particles formed at 15 min. (B) TEM image of Au nanocrystals formed at 36 h, showing the early stage of Au star.

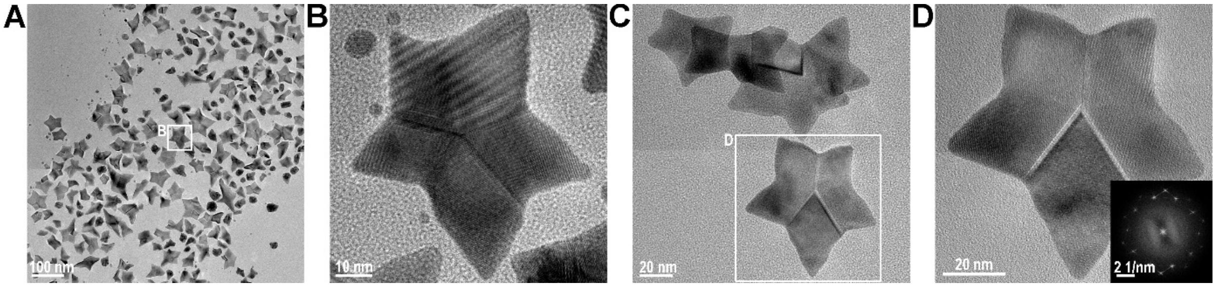

Figure S8. TEM images confirm the presence of five-twinned Au stars even after 9-day incubation.

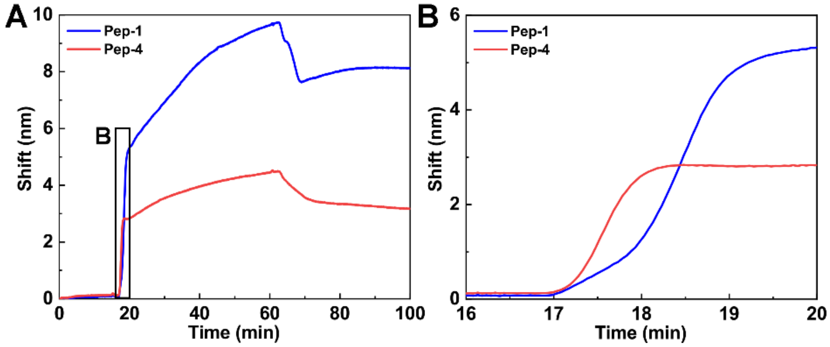

Figure S9. The SPR sensorgrams. The equilibrium SPR shift is higher for pep-1 than pep-4, which means that pep-1 has a higher surface coverage. These observations demonstrated a stronger binding affinitiy of pep-1 than pep-4 on gold film.

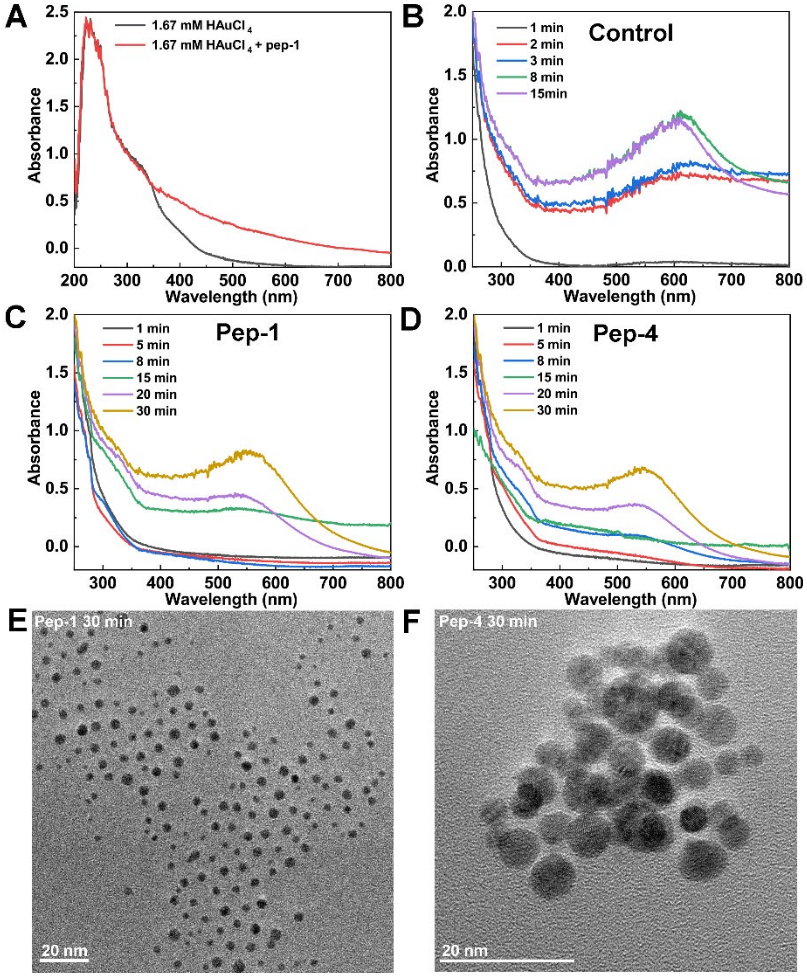

Figure S10. UV-Vis absorption spectra and TEM characterization. (A) $HAuCl_4$ solution and $HAuCl_4$-pep-1 solution. (B-D) In situ UV-Vis spectra shows the formation process of Au nanoparticles without additives and with pep-1 and pep-4. (E-F) The TEM images showing the prepared Au nanocrystals with similar size in the presence of pep-1 and pep-4.

Reference:

1. Zuckermann, R.N.; Kerr, J.M.; Kent, S.B.H.; Moos, W. H. *J. Am. Chem. Soc.* **1992,** 114(26), 10646-10647.

2. Yan, F.; Liu, L.; Walsh, T. R.; Gong, Y.; El-Khoury, P. Z.; Zhang, Y.; Zhu, Z.; De Yoreo, J. J.; Engelhard, M. H.; Zhang, X.; Chen, C.-L. *Nat. Commun.* **2018,** 9 (1), 2327.

3. Velazquez-Salazar, J. J.; Bazan-Diaz, L.; Zhang, Q.; Mendoza-Cruz, R.; Montano-Priede, L.; Guisbiers, G.; Large, N.; Link, S.; Jose-Yacaman, M. *ACS Nano* **2019**, 13 (9), 10113-10128.
